# Five-Year-Old Preschoolers’ Sharing is Influenced by Anticipated Reciprocation

**DOI:** 10.3389/fpsyg.2016.00460

**Published:** 2016-03-31

**Authors:** Mingrui Xiong, Jiannong Shi, Zhen Wu, Zhen Zhang

**Affiliations:** ^1^Key Laboratory of Behavioral Science, Institute of Psychology – Chinese Academy of SciencesBeijing, China; ^2^University of Chinese Academy of SciencesBeijing, China; ^3^Department of Learning and Philosophy, Aalborg UniversityAalborg, Denmark; ^4^Chengdu Normal UniversityChengdu, China; ^5^Department of Psychology, Tsinghua UniversityBeijing, China

**Keywords:** prosociality, strategic behavior, preschooler, direct reciprocity, self-interest

## Abstract

Whether children share in anticipation of future benefits returned by a partner is an interesting question. In this study, 5-year-old children and an adult partner played a sharing game, in which children donated first and the partner donated afterward. In Experiment 1, the partner’s resources were more attractive than the child’s. In the reciprocal condition, the child was told that s/he would be a recipient when the partner played as a donor. In the non-reciprocal condition, however, the child was told that an anonymous child would be the recipient when the partner donated. Results showed that children shared more with the partner when they knew that they would be a recipient later. In Experiment 2, the child was always the recipient when the partner donated, but the partner’s resources were more desirable than the child’s in the high-value condition, and less desirable in the low-value condition. We found that children were more generous when the partner’s resources were valued higher. These findings demonstrate that 5-year-old preschoolers’ sharing choices take into account the anticipated reciprocity of the recipient, suggesting either self-interested tactical sharing or direct reciprocity in advance of receiving. Specifically, they adjust their sharing behavior depending on whether a partner has the potential to reciprocate, and whether it is worth sharing relative to the value of the payback.

## Introduction

Prosocial behavior is widespread in human society (Melis and Semmann, 2010), and has roots early in development, with studies indicating that even toddlers desire to help others or share resources with others (Warneken and Tomasello, 2006; Warneken et al., 2007; Wu and Su, 2014). Apparently, the outcome of prosocial behavior is producing or maintaining the well-being of others (Brief and Motowidlo, 1986). However, the underlying motivations are varied (Leimgruber et al., 2012). Some prosocial behaviors are altruistic, aiming to increase other’s welfare (Warneken and Tomasello, 2007; Hepach et al., 2013); whereas, other prosocial behaviors are “strategic”, using them as a means to achieve another goal, such as benefiting oneself in the long run (Güroǧlu et al., 2009; Steinbeis et al., 2012; Martin and Olson, 2015). This latter kind of prosocial performance is a general ingredient of prosociality in daily life. It has been documented widely in adult studies; however, less is known about how young children perform prosocial behaviors strategically.

There are robust findings that adults strategically perform prosocial behavior out of selfish motivation. For example, they manage their reputation by behaving generously in front of observers (Milinski et al., 2002; Van Vugt and Hardy, 2010; Kanazawa and Fontaine, 2013), because a good reputation can provide priority for future cooperation with others (Nowak and Sigmund, 2005). In addition, adults share more to elicit direct reciprocity, a form of interaction in which individuals do the same thing toward their partner as what their partner did to them (Wilkowski and Chai, 2012). That is, adults are more generous toward a potential direct reciprocator in order to get payback from him/her subsequently (Cox, 2004; Stanca et al., 2009; Kamas and Preston, 2012).

Similar results have been shown in primary school children. A recent study found that 10- and 11-year-old children spontaneously cooperated when there was a high possibility for future interaction (Blake et al., 2015b). This study adopted a paradigm of Prisoner’s Dilemma (PD), in which a child and an anonymous partner decided simultaneously whether to cooperate or to defect. Individuals would maximize their overall payoff if both players cooperated; whereas, individuals would get a higher individual payment if they defected from a cooperative partner. Results showed that, compared with playing with a different partner in every round, children cooperated more if they played with the same partner repeatedly. Therefore, this suggests that 10- to 11-year-old children strategically cooperate when future direct reciprocity is more likely.

Can younger children perform strategic prosocial behavior as well? Some studies on audience effects have provided a positive answer to this question. For example, one study showed that 6-year-old children seemed to have a sense of reputation, pretending to be fair toward others in certain situations (Shaw et al., 2014). In their study, an experimenter either allocated four erasers equally between a participant and a non-present partner (a fair condition), or gave one eraser to the child but two erasers to the partner (an unfair condition). The experimenter then left and came back with an additional eraser, asking whether she should give the eraser to the participant or throw it away. In the unfair condition, however, the participant got another eraser from a second experimenter in private when the first experimenter was outside. Results showed that most 6-year-old children chose to throw away the additional eraser in the fair condition, so that each child remained with two erasers. However, when the first experimenter did not know the existence of that secret eraser from the second experimenter, fewer children chose to throw the additional eraser away because it appeared fair to the first experimenter (Shaw et al., 2014). The audience effects even emerge in 5-year-old children, who behave more generously when others are aware of their action (Leimgruber et al., 2012), especially when the observer has an opportunity to share resources with them later (Engelmann et al., 2013). Moreover, a recent study suggested that the audience effects in preschoolers was driven by a concern for direct responses from real partners, rather than a mere sense of being monitored (Fujii et al., 2015). In this study, 5-year-old children shared more when their behavior was monitored by a real person than by flowers shown on a computer; however, being monitored by staring eyes on the computer did not increase children’s sharing compared to being monitored by a picture of flowers. These above studies showed that children could share strategically to actively impress a third party, who might help them gain in the future or who might give them direct punishments or reward; however, less is known about children’s strategic behavior to impress a potential direct reciprocator, who has a chance to reciprocate the previously received benefits. Because a face-to-face interaction is very common in daily life, it is important to explore strategic prosocial behavior during social interactions. Therefore, the aim of the present study is to explore whether preschoolers can perform prosocial behavior strategically in anticipation of payback from a partner with whom they directly interact.

Two recent studies have provided some evidence for children’s strategic prosocial behavior during a face-to-face interaction. Kuhlmeier et al. (2014) proposed that preschoolers might be able to choose a partner based on whether the partner was able to repay their initial prosocial actions. This view was recently supported by Kenward et al.’s (2015) research utilizing a three-player sharing game, in which children and two other players could exchange tokens for candies in turn. Participants exchanged for candies first and they always got two candies. Then, participants were asked to choose between a rich partner and a poor partner with whom to share candies. The rich partner had many tokens, thus could exchange for many candies later. By contrast, the poor one had no tokens, thus had no opportunity to get candies. Although, both partners stated that they would share with the one who shared with them before if they get candies, only the rich partner was clearly able to reciprocate during the test. Results showed that 4-year-old children were more likely to share with the rich partner (Kenward et al., 2015). This study demonstrated that children strategically chose a partner who had the capacity to return favors. In this study, however, participants were forced to choose one person to share with and the reciprocity norm was explicitly verbalized. Such a design could not provide information about whether children would share when giving was optional. In addition, explicit emphasis of the reciprocity norm might have promoted children’s strategic sharing. Therefore, it remains an open question whether children share based on their initiative in anticipation of reciprocity, when ostensive cues from the environment are absent.

A similar study conducted by Sebastián-Enesco and Warneken (2015) could partly answer this question. In this study, they explored sharing behavior in 3- and 5-year-old children when a puppet partner was either able or unable to reciprocate. In an experimental condition, a child and a puppet partner received some balls that could be used to play on two machines, one of which was more interesting than the other one. Only the participant got some balls when playing on the less interesting machine; afterward, only the puppet got some balls when playing on the more interesting one. At the beginning of the game, the experimenter told participants that the child would decide first whether to share balls with the puppet when playing on the less attractive machine, and then the puppet would decide whether to share with the child when playing on the more attractive machine. In fact, the puppet shared in a tit-for-tat way. Such a process of taking turns to decide whether to share went on for four rounds. In the control condition, the puppet did not have a chance to share resources. There was just a less interesting machine and only the participant received some balls to play. After the child made a sharing decision with the puppet, they played a drawing game together. The study focused on whether participants shared differently in the two conditions. They found that 5-year-olds, but not 3-year-olds, shared more balls in the experimental condition in which the child and the puppet took turns sharing.

This study suggested that 5-year-old children shared strategically based on whether a partner had the opportunity to repay the initial prosocial act. However, due to the study design of repeated interactions, it was not clear whether children strategically shared at the very beginning or they gradually learned to share strategically resulting from the partner’s tit-for-tat reciprocity strategy. In other words, children’s sharing behavior might be influenced by their partner’s *previous* reactions. To exclude the effect of partner’s previous reactions, in the current study, children and the partner (a female adult) switched roles only once, with children sharing first and the partner sharing later. We could thus focus on whether children share in anticipation of *future* benefits out of self-interest consideration without depending on any external cues or prior interactions. In addition, in Sebastián-Enesco and Warneken’s (2015) control condition, the child and the puppet played a drawing game together, in which no balls were used. One possibility might be that children shared more in the experimental condition because they knew the puppet needed balls later, but not in the control condition. To rule out this possibility, in the current study, stickers were used in both conditions. Children donated stickers first, and then the partner donated stickers either with the child participant in the reciprocal condition or with an anonymous child in the non-reciprocal condition. We aimed to investigate whether children would share differently when the partner would either pay them back, or when the partner would share with someone else.

In addition, if children’s strategic sharing is due to selfish motivation, it is suggested that they can not only distinguish whether the recipient has an opportunity to reciprocate, but also be sensitive to the necessity of obtaining a possible payback. In Experiment 2, we thus further tested whether 5-year-old children adjusted their sharing behavior depending on whether it is worth eliciting reciprocity. We varied the attractiveness (and thus the value) of the partner’s possession. A vast literature has demonstrated that preschoolers are concerned about resource value. For example, they can judge the relative value of different resources (Harbaugh et al., 2001; Benenson et al., 2015). Preschoolers also took resource value into consideration when distributing resources between two recipients (Shaw and Olson, 2013). Additionally, children from age three modified their own sharing behavior according to the resource value, sharing more when resources were less desirable (Birch and Billman, 1986; Blake and Rand, 2010). Therefore, it is possible that children are also sensitive to their partner’s resource value during the interaction. When the partner’s resources are of higher value than children’s own (thus desirable for children), children may strategically share more in order to elicit the partner’s payback. Similarly, if the partner’s resources are of lower value than children’s, they may be less likely to share because they are less motivated to get payback. To test this possibility, we manipulated the value of the partner’s resources in Experiment 2. Similar to Experiment 1, both players played the sharing game, with the partner playing as a donator after the participant. This time, however, the partner shared with the participant in both conditions, thus the partner always had an opportunity to repay the participant’s favor. The important manipulation was that the partner’s resources were more attractive than the participant’s in one condition and less attractive than the participant’s in the other condition. Thus, by manipulating the partner’ resource value, we aimed to determine if children were able to further judge the necessity of performing a strategic sharing behavior.

To sum up, the current study examined two issues about strategic sharing during a face-to-face interaction. First, we tested whether 5-year-old preschoolers increased sharing when a partner had a potential opportunity to reciprocate and when explicit information about a reciprocity norm was absent. We hypothesized that children would share more resources when they would potentially get a payback. Second, we investigated whether children at this age could adjust their sharing behavior depending on the value of the partner’s available resources that might be reciprocated. We predicted that children would behave generously only when the potential payback was desirable.

## Experiment 1

### Participants

Sixty 5-year-old preschoolers from kindergartens in Beijing, China, participated in this study. Their age ranged from 5 years 1 month to 5 years 11 months (30 females, *M* = 65.45 months, *SD* = 3.42 months). Most of them came from middle-class families. An additional four children were tested, but excluded from analysis because of interruption (*n* = 1), misunderstanding of the task (*n* = 1) and an inconsistent evaluation about material stickers (*n* = 2, for more details, see Design and Procedure). The informed consent was obtained from the parents of all children who participated in the study. The research was approved by the Institutional Review Board of the Institute of Psychology – Chinese Academy of Sciences, and was in accordance with its guidelines.

### Materials

The partner got eight different cartoon stickers, portraying a princess image for female participants and a warrior image for male participants. By contrast, participants received eight different number stickers with number symbols on. Two envelopes in different colors (brown and white) were used to distribute the stickers.

### Design and Procedure

Participants were tested individually in a quiet room in their kindergarten. The experiment was conducted by a female experimenter, who introduced the game rules and was in charge of the course of the experiment. A female assistant, who was blind to the purpose of the study, acted as the participants’ partner. During the test, the participant sat across a table from the partner, and the experimenter sat by one side of the table at a 90° angle to the child and the partner. The procedure had three phases: warm-up, sticker-assignment, and sharing. The whole process was videotaped.

A warm-up phase went first to acclimate children to the new environment and to the partner, during which the partner and the child introduced themselves. Then the experimenter showed them 16 stickers (eight cartoon stickers and eight number stickers) and asked the participant which kind of stickers s/he preferred. To ensure that stickers allocated by all participants were in the same kind and had the same subjective value, we continued the experiment only in participants who ranked cartoon stickers as more attractive. In fact, only two children ranked cartoon stickers as less attractive, and their data were thus excluded from the data analysis. Then, the experimenter asked participants to count each kind of sticker, respectively. There were two purposes for this. One was to ensure that participants could count to eight correctly. The other one was to make sure that participants knew the exact number of each kind of sticker. Participants were corrected immediately once they miscounted and all participants could count to eight after being corrected.

The sticker-assignment phase followed the warm-up phase. The child participant always received less attractive stickers (i.e., number stickers) whereas the partner received more attractive ones (i.e., cartoon stickers). This manipulation aimed to motivate children to share in anticipation of a payback. To ensure that the manipulation occurred naturally, the participant and the partner drew straws to decide which kind of stickers they would get. The rule was that people who drew a card written Arabic numeral 1 would get number stickers. Actually, there was “1” on both cards, and children were asked to draw a card first. As a result, children always received number stickers. Then, children and the partner signed their names on their own envelopes (a brown one and a white one) delivered by the experimenter. The color of the participant’s envelope was counter-balanced across participants.

**FIGURE 1:**
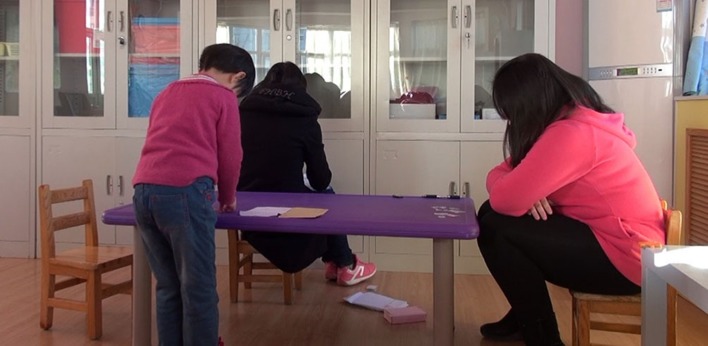
**A participant walked to the middle area to allocate stickers between herself and the partner (in red clothes), with the experimenter (in black clothes) turning her back around**.

After the partner and the participant received their own stickers, the sharing phase began. We employed a between-subjects design. Participants were randomly assigned to either the reciprocal or the non-reciprocal condition. In the reciprocal condition, the experimenter told participants that these number stickers belonged to them, and they could decide whether to share some with their partner. They could either give out any number of stickers, or keep all stickers with them if they did not want to share. They should put the shared stickers into the partner’s envelope and the rest into their own envelope (**Figure 1**). After the game, they would take their envelopes containing the stickers home. Then, the experimenter also told the partner that the cartoon stickers belonged to her, and she would decide how to share after the participant. The procedure was the same in the non-reciprocal condition except that the recipient was an anonymous child other than the child participant when the partner shared. The anonymous child was not present, and was described as a child from another kindergarten who was the same age and gender as the participant.

Before the participant shared, a series of comprehension questions were asked in both conditions to make sure that participants (1) knew who would get the stickers allocated by them and by the partner; (2) knew they would share prior to the partner. If children answered incorrectly, then the rules would be explained again. All participants answered the comprehension questions correctly either the first time or after a repetition of rules. To ensure that children’s sharing behavior would not be influenced by the experimenter, the experimenter told them that during the game she would turn around, and thus could not see how they allocated the stickers. The participant and the partner were not allowed to talk during the game, in case a conversation between them would influence the participant’s initial decision. The partner kept a neutral expression during the whole process and shared half of her resources in both conditions when it was her turn to choose whether to share.

After the game, the experimenter asked who would take the stickers in the white and brown envelopes, respectively. All participants answered the post-experiment questions correctly. In case of possible influence caused by gossip with subsequent participants, children were required to keep the game a secret after getting back to the classroom.

### Coding

The assistant counted the number of stickers shared by the participant after s/he left. She then put the stickers back in the envelope and recorded the number that was shared. The experimenter double-checked the total 1 day later. Interrater reliability reported as Cohen’s κ was 1.00.

### Results and Discussion

**FIGURE 2:**
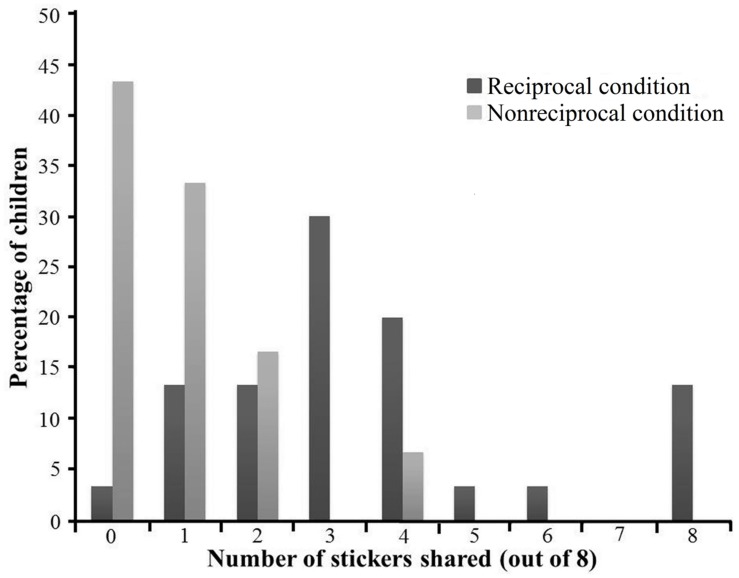
**Distribution of number of stickers shared in Experiment 1 by condition**.

**FIGURE 3:**
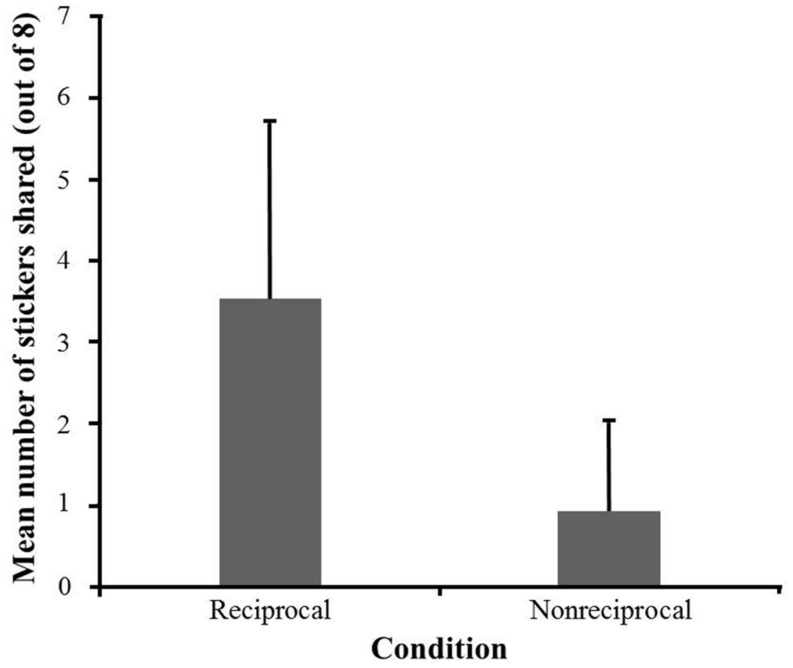
**The mean number (and standard deviation) of stickers shared in the two conditions in Experiment 1**.

The data were analyzed using SPSS 20.0 (IBM, Inc., Armonk, NY, USA). The distributions of participants’ sharing in the two conditions are illustrated in **Figure 2**. We first tested whether the proportion of participants who shared differed between the two conditions. A Fisher’s exact test revealed a significant condition effect, *p* < 0.001, with more participants shared in the reciprocal condition (29 out of 30 participants) than in the non-reciprocal condition (17 out of 30 participants). Then, we tested whether there was a difference in the mean number of stickers shared between the two conditions. The average number of stickers shared in the two conditions is shown in **Figure 3**. A 2 (Condition: reciprocal condition vs. non-reciprocal condition) × 2 (Gender: boys vs. girls) analysis of variance (ANOVA) was conducted with the number of stickers shared as the dependent variable. The result showed a significant main effect of condition, *F*(1,56) = 32.71, *p* < 0.001, $\eta_{p}^{2}$ = 0.37, with more resources shared in the reciprocal condition (*M* = 3.53, *SD* = 2.19) than in the non-reciprocal condition (*M* = 0.93, *SD* = 1.11). The main effect of gender was not significant, *F*(1,56) = 0.02, *p* = 0.88, $\eta_{p}^{2}$ < 0.001, nor was the interaction of gender × condition, *F*(1,56) = 0.54, *p* = 0.47, $\eta_{p}^{2}$ = 0.01.

Our results revealed that more 5-year-old children shared in the reciprocal condition, and they also shared more stickers in the reciprocal condition than in the non-reciprocal condition. In the reciprocal condition, children knew there was a chance that the partner would reciprocate the favor; in the non-reciprocal condition, however, children knew that the partner would not have a chance to reciprocate. Accordingly, children behaved more generously toward the potential reciprocator. Therefore, these results suggest that children perform the prosocial action in the reciprocal condition strategically to elicit reciprocity for self-interest.

However, there is another possible explanation for the above results. Children may have shared more in the reciprocal condition just because the partner would play a game with them later rather than with others. That is, children’s generosity had nothing to do with their concern for self-interest, but was simply a response in some general way to their involvement in the iterative game (a “sharing game” in the current study). To rule out this confound, in Experiment 2, the partner later played a “sharing game” with the child in both conditions. Moreover, we manipulated the value of the resources possessed by the partner to further explore whether 5-year-old children could differentiate the necessity of eliciting the partner’s payback. If children in Experiment 1 shared more in the reciprocal condition just because the partner would play a game with them later, then they would share the same amount regardless of the different conditions in Experiment 2 because the partner in both conditions would play a game with them instead of with others. Conversely, if children indeed performed prosocial behavior to increase their own possible payback, they would be less generous when the partner’s possession was less attractive.

## Experiment 2

### Participants

Fifty-nine 5-year-old preschoolers from kindergartens in Beijing participated in this study. Their age ranged from 5 years 1 month to 5 years 11 months (33 females, *M* = 66 months, *SD* = 3.12 months). Most of them came from middle-class families. An additional three participants were tested, but excluded from analysis because of not understanding the task (*n* = 2) and assistant error (*n* = 1). Informed consent was obtained from the parents of all children who participated in the study.

### Materials

Participants got eight different number stickers as in Experiment 1. The partner’s stickers were either eight different cartoon stickers portraying a princess image for girls and a warrior image for boys (considered as “high-value” stickers) or eight different number stickers just as the participants’, but much smaller in size (considered as “low-value” stickers). Other materials were the same as in Experiment 1.

### Design and Procedure

Participants were randomly assigned to the high-value and the low-value conditions, which differed according to the kind of stickers possessed by the partner. In fact, the partner’s stickers were more attractive (i.e., more interesting content, princess, or warrior) than the child’s in the high-value condition, but less attractive (i.e., smaller size) than the child’s in the low-value condition. The procedure in both conditions was the same as that in the reciprocal condition in Experiment 1, with participants playing as the donator first, and they switched roles subsequently. A video camera recorded the process.

A pretest was run to ensure that the partner’s stickers were more attractive than the participants’ in the high-value condition; and less attractive than the participants’ in the low-value condition. Twenty-eight 5-year-old preschoolers from the same kindergarten participated in the pretest. Consistent with our anticipation, most of them, 20 out of 28, thought cartoon stickers were more attractive than number stickers (binomial test, *p* = 0.036), and 26 out of 28 children thought number stickers that were large were more attractive than those that were small (binomial test, *p* < 0.001). This result confirmed that the stickers’ value assumed in different conditions was creditable.

### Coding

The procedure of coding was the same as that in Experiment 1. Interrater reliability reported as Cohen’s κ was 1.00.

### Results and Discussion

**FIGURE 4:**
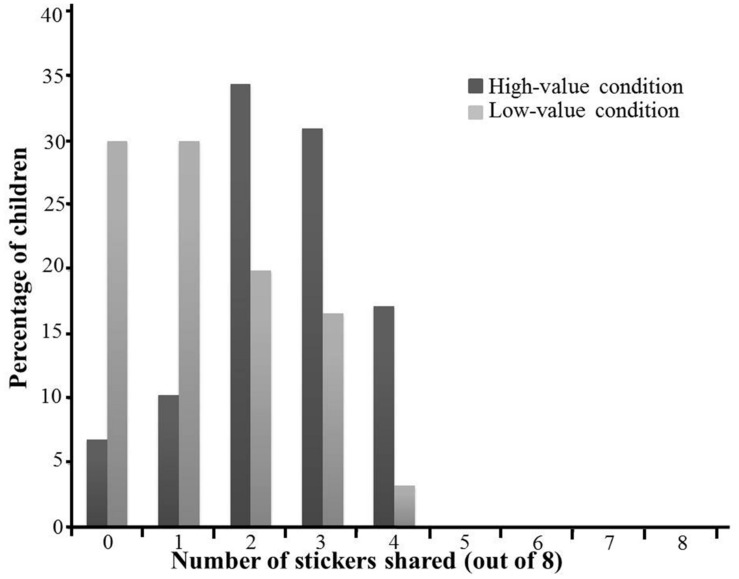
**Distribution of number of stickers shared in Experiment 2 by condition**.

**FIGURE 5:**
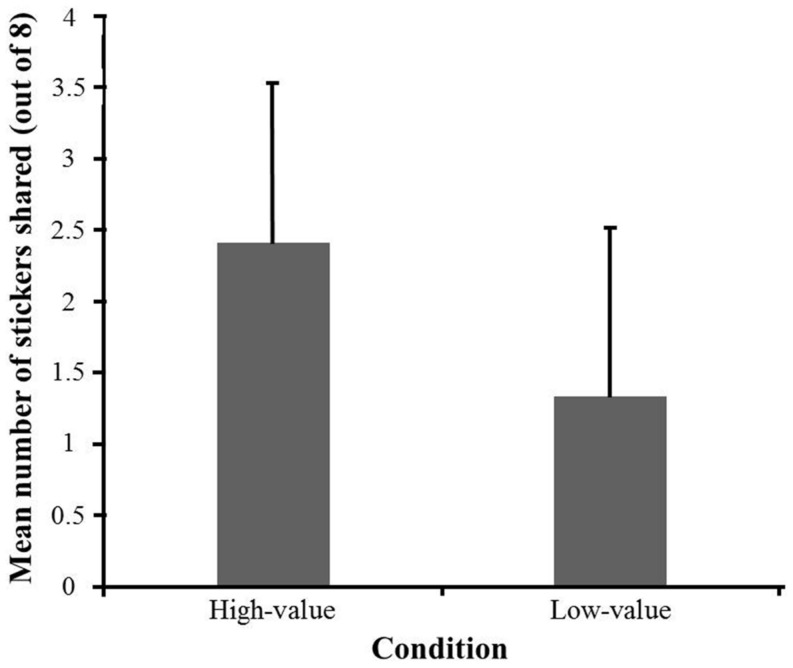
**The mean number (and standard deviation) of stickers shared in the two conditions in Experiment 2**.

**Figure 4** illustrated the distribution of stickers shared in both the high-value and the low-value conditions. We first analyzed whether the percentage of participants who shared differed between the two conditions. A Fisher’s exact test revealed that children were more likely to share in the high-value condition (27 out of 29 participants) than in the low-value condition (21 out of 30 participants), *p* = 0.042. Next, we analyzed whether there was a difference in the mean number of shared stickers between the two conditions (**Figure 5**). A 2 (Condition: high-value condition vs. low-value condition) × 2 (Gender: boys vs. girls) ANOVA was conducted with the number of stickers shared as the dependent variable. Results revealed a significant main effect of condition, *F*(1,55) = 11.55, *p* = 0.001, $\eta_{p}^{2}$ = 0.17, with participants sharing more stickers in the high-value condition (*M* = 2.41, *SD* = 1.12) than that in the low-value condition (*M* = 1.33, *SD* = 1.18). There were no significant results of either the main effect of gender, *F*(1,55) = 0.032, *p* = 0.86, $\eta_{p}^{2}$ = 0.001, or the interaction of gender and condition, *F*(1,55) = 1.46, *p* = 0.23, $\eta_{p}^{2}$ = 0.026.

To figure out whether the partner’s involvement in the iterative game had any influence on children’s sharing, we further compared the proportion of children who shared, as well as the mean number of stickers they shared between the low-value condition in Experiment 2 and the non-reciprocal condition in Experiment 1. A Fisher’s exact test revealed that the percentage of participants who shared did not differ significantly between the low-value (21 out of 30 participants) and the non-reciprocal condition (17 out of 30 participants), *p* = 0.42. Meanwhile, an independent *t*-test revealed that the mean number of shared stickers between the low-value condition (*M* = 1.33, *SD* = 1.18) and the non-reciprocal condition (*M* = 0.93, *SD* = 1.11) was not significantly different either, *t*(58) = 1.35, *p* = 0.13.

Results showed that 5-year-old children shared more stickers in the high-value condition than in the low-value condition. This finding suggests that apart from detecting the potential opportunity for a partner to reciprocate, 5-year-old children could further recognize the necessity to elicit a partner’s reciprocity. They shared more when the partner’s resources were more attractive than theirs, compared to when the partner’s resources were less attractive. Moreover, we didn’t find that children shared differently between the non-reciprocal condition in Experiment 1 and the low-value condition in Experiment 2, which further excluded the possible explanation that children shared more simply because the partner would play a game with them later. Thus, these findings together suggest that children may behave prosocially out of self-benefiting motivation.

## General Discussion

The current study investigated whether children shared strategically based on selfish motivation in a direct reciprocity situation. Experiment 1 demonstrated that 5-year-old children shared more resources when a partner had a chance to reciprocate with them, compared to when such a chance did not exist. It suggests that children perform prosocial behavior in the reciprocal condition strategically to benefit themselves eventually. In addition to adjusting prosocial behavior according to the existence of a reciprocal opportunity, children could further vary prosociality depending on the value of the resources that might be reciprocated. As shown in Experiment 2, when the opportunity of getting a payback existed in both conditions, children shared more when the partner’s resources were more desirable than less desirable. Together, both Experiment 1 and Experiment 2 suggest that 5-year-old children could strategically share to elicit reciprocity, aiming to increase their own welfare (although see below for an alternative explanation in terms of reciprocity in advance of receiving).

These results are consistent with previous findings showing that children’s prosocial behavior is influenced by an anticipation of reciprocity; more importantly, it reduces possible influences from external cues about reciprocity. For example, a very recent study demonstrated that children preferred a partner who had a chance to interact with them later and verbally expressed that s/he would reciprocate (Kenward et al., 2015). However, this study did not show whether children could choose a reciprocal partner when such explicit cues about reciprocity (e.g., the partner’s verbal expression of reciprocity norm) do not exist. Additionally, research also showed that children were more generous in a repeated interactive situation, where the partner reacted with a tit-for-tat strategy (Sebastián-Enesco and Warneken, 2015). However, in this study it was hard to tell apart whether children strategically shared at the very beginning or they learned how to share strategically from the partner’s prior reactions. The current study eliminated the possible influence from external cues. The partner did not explicitly stress the reciprocity norm; additionally, a one round interaction between the participant and the partner ruled out the possibility that participants learned how to react based on the partner’s previous feedback. Without these external cues, we still found that children shared more when the potential for direct reciprocity was possible. These findings thus provide strong evidence that children initiatively adjust their sharing in anticipation of future benefits.

In addition, our results further demonstrate that children not only share more when a reciprocal opportunity exists, they also share more when it is more worth it to elicit potential reciprocity. In Experiment 2, when children donated first and then switched roles to be a receiver with a partner, 5-year-old children were more willing to make a costly donation when the interactive partner possessed valuable resources, thereby exhibiting a strong self-benefiting motivation. By showing that children take the value of their partner’s potential payback into account when sharing, our results thus align with previous findings that children are concerned about their partner’s knowledge state about their choices during an interaction (Leimgruber et al., 2012). In this prior study, 5-year-old children had to decide whether to deliver one or four stickers to a partner at no cost to themselves. The donation options were either obscure or visible to the partner. Children were more likely to deliver four stickers when the partner was aware of the allocation options than when the partner was not aware. Besides distinguishing whether a partner was aware of their choices, the current study further demonstrated that 5-year-old children were concerned about whether a partner’s resources were of high or low value. Accordingly, children appeared to be more generous when the partner’s payback could increase the value of their prize. Together, the combined results from the above studies demonstrate that children are more generous when their anticipated gains could enrich their overall gain, either in the long run (Leimgruber et al., 2012) or in the near future (Kenward et al., 2015; also shown in the current study).

Our study thus demonstrates the existence of strategic prosocial behavior in 5-year-old children, which extends previous finding based on Western, educated, industrialized, rich and democratic (WEIRD) populations to Chinese culture. It seems that strategic sharing behavior in children is common in different cultures, though a large number of studies have shown that there is substantial variability in children’s social and economic behavior across countries (Henrich et al., 2010; Blake et al., 2015a). It would be interesting for future studies to investigate whether the level of children’s strategic sharing and its underlying mechanisms vary across cultures.

In addition to demonstrating the existence of strategic sharing behavior in a non-WEIRD sample, the current study shows preschoolers’ strategic sharing with a paradigm of Dictator Game (DG, one decides how much to give and the recipient is bound to accept the offer, see also Kenward et al., 2015; Sebastián-Enesco and Warneken, 2015). It thus supplements previous studies indicating children’s strategic behavior with other paradigms, such as PD and Ultimatum Game (UG). For example, Blake et al. (2015b) found that 10- to 11-year-old children were more cooperative in a game of PD when they repeatedly interacted with the same partner, compared to when interacting with a different partner in every round; moreover, this difference persisted even when researchers only took the first round of play into consideration. It suggests that children cooperate strategically and spontaneously when it is possible for future direct reciprocity. Thus, both the findings in the current DG and the prior studies utilizing PD demonstrate that children are strategically prosocial when they can benefit from the potential positive reciprocity.

Moreover, previous research has also found that children tactically share more when they may suffer a loss from the possible negative reciprocity, where unkindness always returns unkindness (Narotzky and Moreno, 2002). Strategic sharing to avoid possible loss caused by negative reciprocity is reflected in the UG (Bereby-Meyer and Fiks, 2013). In the UG, two participants take the part of the donor and the recipient. The donor decides how much to share with the recipient. If the recipient accepts the allocation, the resources would be divided as proposed by the donor; if the recipient rejects the allocation, both of them would receive nothing. Dissimilar from the UG, the recipient in the DG has no opportunity to impose a loss on the donor, and thus negative reciprocity is impossible. Comparing the allocation results from both the UG and DG, researchers found that children were more generous as a proposer in the UG than in the DG (Bereby-Meyer and Fiks, 2013). Even 4-year-old preschoolers showed this tendency, with a 7% larger proposal in the UG (Lucas et al., 2008). The much more sharing in the UG suggests that children adjust their distribution to avoid possible loss due to the threat of potential negative reciprocity. Together with previous studies, our findings show that children weigh their gains and losses when they share, behaving generously if they expect to get a desirable payback, or if they want to avoid an undesirable outcome.

However, apart from strategically eliciting reciprocity, there may be other explanations for children’s generosity in our study when the partner had the potential to reciprocate desirable resources. One may think that children shared more in the high-value condition just because they were frustrated by their partner’s desirable resources, and then lost interest in their own resources. This interpretation is unlikely, because children did not increase their sharing when the partner also possessed more attractive stickers in the non-reciprocal condition in Experiment 1. Therefore, the more generous behavior in the high value condition cannot be attributed to frustration when the partner had more attractive resources.

Another possibility is that sharing more in the reciprocal condition and in the high-value condition was due to children’s direct reciprocity in advance of receiving. That is, an anticipation of receiving from the partner prompted children to direct reciprocate themselves in advance of receiving in the reciprocal condition in Experiment 1. Similarly, children in Experiment 2 might also wish to directly reciprocate (in advance), with the value of their reciprocal act being adjusted to the value of the items they expected to receive and therefore resulted in sharing more in the high-value condition than in the low-value condition. Sharing for direct reciprocity in advance is an interesting yet unexplored topic; therefore, future research is required to investigate whether strategic sharing for self-interest, direct reciprocity in advance or the combination of both contribute to children’s generosity when they could benefit from the partner’s potential reciprocity.

Furthermore, another interesting question about children’s generosity in our study is to what extent this behavior is truly self-benefiting on a mechanistic and not just a functional level. Are children really thinking about how to behave to their advantage, or is the process more automatic? Although the results showed that children shared more when the partner had a chance to reciprocate and when there were benefits to elicit the partner’s reciprocity, the design in the current study could not provide convincing evidence about the process of decision-making underlying children’s sharing. Future study could examine this issue by manipulating the time that allows children to make decisions when they share with a possible reciprocator. Researchers have proposed that thought-out responses require additional time for deliberation (Rand et al., 2012). Therefore, setting a time constraint when children share can help detect its underlying processes. If children share more when their decision is free of time pressure than when it is under time pressure, a stronger conclusion may be drawn that children’s generosity is a thought-out behavior rather than an unconscious response.

It is noteworthy that children also shared some, though less, in the non-reciprocal condition in Experiment 1, when the partner had no opportunity to reciprocate. This suggests that children’s sharing may not be driven purely by a motivation to maximize their own profit; otherwise they would not have shared at all in the non-reciprocal condition, when sharing reduces the number of their own stickers and there is no chance to get them back. Giving out resources when no obvious gains are foreseen has also been observed in prior studies, in which preschoolers often share stickers with anonymous others who have no chance to reciprocate (Benenson et al., 2007; Harbaugh and Krause, 2007; Gummerum et al., 2010) and its underlying mechanisms maybe empathy or other-regarding preference (Warneken and Tomasello, 2009; Edele et al., 2013). However, children’s sharing in the non-reciprocal condition in the current study can hardly be explained by these mechanisms, because the partner owns more desirable resources than children themselves. In terms of the underlying mechanism of sharing behavior directed toward wealthier partners when there is no reciprocity, is it because of reputation concern (Engelmann et al., 2012; Leimgruber et al., 2012), a motivation of socialization to establish a good relationship with others (Brownell, 2013; Brownell et al., 2013), or a generalized reciprocity consideration (Rankin and Taborsky, 2009; Gray et al., 2014)? Future research is needed to explore these possibilities. These results thereby illustrate that the motivation behind prosociality is various, and may be a combination of both altruistic and selfish motivations.

In sum, the current study shows that preschoolers initiatively conduct a prosocial behavior oriented at future payback. Besides “looking back” when making decisions about how much to share (Robbins and Rochat, 2011; House et al., 2013; Warneken and Tomasello, 2013), our finding expands this line of research by showing that 5-year-old children also “look ahead”, linking their current sharing behavior with their partner’s possible subsequent decisions. They behave generously with an expectation that their kindness would be returned by kindness through a partner’s subsequent moves. In particular, children did not know for sure about the partner’s actual sharing when they made their own decisions, yet they shared when there was an opportunity for reciprocity, and when the potential reciprocal resources were desirable. The current study thus suggests that 5-year-old children’s prosocial behavior can go beyond a reaction to their partner’s prior kindness (Robbins and Rochat, 2011; House et al., 2013; Warneken and Tomasello, 2013), and can be further motivated by an anticipation of future benefits returned from the partner. It would be interesting for future studies to investigate the origins of these “look ahead” prosocial behaviors, and the underlying mechanisms, such as delay of gratification, prospection, theory of mind, etc. (Moore and Macgillivray, 2004; Coricelli and Nagel, 2009; Brandimonte et al., 2010; Takagishi et al., 2010; Garon et al., 2011).

## Author Contributions

MX, ZZ, and ZW contributed to the design of the research; JS and ZZ recruited the participants; MX conducted the experiment and analyzed the data; JS and ZZ supervised the project; ZZ, ZW, and JS gave valuable comments about the interpretation of the data; MX, ZW, and ZZ all contributed to the writing of the manuscript; all authors reviewed the manuscript for important content.

## Conflict of Interest Statement

The authors declare that the research was conducted in the absence of any commercial or financial relationships that could be construed as a potential conflict of interest.
